# Tumor-Derived Syndecan-1 Mediates Distal Cross-Talk with Bone that Enhances Osteoclastogenesis

**DOI:** 10.1002/jbmr.16

**Published:** 2010-01-14

**Authors:** Thomas Kelly, Larry J Suva, Kristy M Nicks, Veronica MacLeod, Ralph D Sanderson

**Affiliations:** 1 Department of Pathology, Winthrop P Rockefeller Cancer Institute, University of Arkansas for Medical SciencesLittle Rock, AR, USA; 2 Center for Orthopedic Research, Departments of Orthopedic Surgery and Physiology and Biophysics, Winthrop P Rockefeller Cancer Institute, University of Arkansas for Medical SciencesLittle Rock, AR, USA; 3 Department of Pathology, Center for Metabolic Bone Disease, UAB Comprehensive Cancer Center, University of Alabama at BirminghamBirmingham, AL, USA

**Keywords:** CD138, cytokines, heparan sulfate proteoglycans, bone metastasis, tumor microenvironment, growth factors

## Abstract

Tumor-stimulated bone resorption fuels tumor growth and marks a dramatic decline in the health and prognosis of breast cancer patients. Identifying mechanisms that mediate cross-talk between tumor and bone remains a key challenge. We previously demonstrated that breast cancer cells expressing high levels of heparanase exhibit enhanced shedding of the syndecan-1 proteoglycan. Moreover, when these heparanase-high cells are implanted in the mammary fat pad, they elevate bone resorption. In this study, conditioned medium from breast cancer cells expressing high levels of heparanase was shown to significantly stimulate human osteoclastogenesis in vitro (*p* < .05). The osteoclastogenic activity in the medium of heparanase-high cells was traced to the presence of syndecan-1, intact heparan sulfate chains, and heat-labile factor(s), including the chemokine interleukin 8 (IL-8). The enhanced osteoclastogenesis promoted by the heparanase-high cells results in a dramatic increase in bone resorption in vitro. In addition, the long bones of animals bearing heparanase-high tumors in the mammary fat pad had significantly higher numbers of osteoclasts compared with animals bearing tumors expressing low levels of heparanase (*p* < .05). Together these data suggest that syndecan-1 shed by tumor cells exerts biologic effects distal to the primary tumor and that it participates in driving osteoclastogenesis and the resulting bone destruction. © 2010 American Society for Bone and Mineral Research.

## Introduction

Bone metastasis is a frequent, often debilitating, and painful complication of breast cancer,(1) yet the mechanisms by which breast cancer cells colonize bone to form metastases remain poorly understood. A number of molecules elaborated by breast cancer cells, including parathyroid hormone–related peptide (PTHrP),(2) interleukin 8 (IL-8),(3) IL-11,(4) and many others, have been shown to stimulate osteoclastogenesis and the resulting bone destruction.(5)

Once osteoclasts are activated to resorb bone, growth factors that are bound to the bone matrix and that can stimulate metastatic tumor cell growth are released.(6) This positive-feedback loop produces a microenvironment within the bone marrow that has the potential to sustain tumor growth.(5) The cross-talk between the breast cancer cells colonizing bone and the resident osteoclasts (and their precursors) is believed to be particularly important for the initial establishment of bone metastases, at least in animal models.(6) For example, in various mouse models, treatment with bone-resorption-inhibiting bisphosphonates reduces the number of bone metastatic foci if administered prior to the arrival of the tumor cells in bone.(7) However, bisphosphonates do not inhibit the growth of metastatic foci once they are established in bone.(7) Thus, although bisphosphonates maintain bone density in breast cancer patients with bone metastases and have been shown to increase disease-free survival in premenopausal patients with estrogen-responsive early breast cancer,(8) in animal models, bisphosphonates do not slow the growth of established lesions. This suggests that after becoming established, bone metastases may no longer require active bone resorption.(7) The question of how osteoclastogenesis and bone resorption are stimulated initially by tumor cells prior to and after their arrival in the skeleton remains largely unanswered.

Heparanase is an enzyme that acts both at the cell surface and within the extracellular matrix to degrade polymeric heparan sulfate chains into shorter-chain-length, biologically active oligosaccharides of 18 to 20 sugar residues.(9) In human breast cancer, heparanase overexpression has been associated with increased metastasis and poor prognosis.(10) Heparanase may contribute to disease progression through its well-established proangiogenic effects that drive vessel formation and fuel explosive tumor growth.(11–14) In addition to stimulating tumor growth,(11,12,15–17) heparanase has been implicated in local invasion and metastasis(18,19) and is upregulated by estrogen.(20)

One of the major heparan sulfates expressed on the surface of human breast cancer cell lines and human breast tumors is syndecan-1.(21,22) Syndecan-1 is a transmembrane (type I) heparan sulfate proteoglycan known to regulate multiple biologic functions, including cell–extracellular matrix adhesion, and it has a well-documented and important role as a growth factor coreceptor.(23) In addition, syndecan-1 on the surfaces of cells can activate cell-surface integrins, thereby promoting breast tumor cell spread, migration, and angiogenesis.(24,25) In breast cancer patients, syndecan-1 is upregulated in the reactive stroma adjacent to malignant tumor cells(26) and is highly abundant in the bone marrow stroma of patients with multiple myeloma.(27) In fact, the shedding of syndecan-1 from cells within primary human breast tumors has emerged as an important regulator of the tumor microenvironment, where syndecan-1 is associated with enhanced tumor proliferation, angiogenesis, and the activity of matrix metalloproteinases (MMPs).(28) When shed from the cell surface, syndecan-1 can facilitate the growth, angiogenesis, and metastasis of tumors.(29,30) Importantly, the functional activities of syndecan-1 can be modulated by the enzymatic activity of heparanase, which specifically cleaves heparan sulfate chains.(31)

Previously we demonstrated that bone resorption occurs before the appearance of overt bony metastases in animals bearing breast tumors overexpressing heparanase.(12) Subsequently, we also demonstrated that breast cancer cells overexpressing heparanase release elevated levels of syndecan-1 into the growth medium(29) and, moreover, that the overexpression of catalytically inactive heparanase did not increase the shedding of syndecan-1.(29) These data suggest that factors may be released into the systemic circulation by breast cancer cells overexpressing heparanase that may stimulate distant osteoclastogenesis and the subsequent bone destruction.(12) In the studies described here, increased osteoclast numbers and bone resorption were observed in the long bones of animals bearing human breast tumor mammary fat pad xenografts overexpressing active heparanase. In addition, in vitro osteoclastogenesis assays demonstrated that shed syndecan-1 was required for the enhanced osteoclastogenic activity of the tumor cells. These findings identify syndecan-1 that is shed by tumor cells expressing heparanase as a potent pro-osteoclastogenic agent.

## Materials and Methods

### Cell culture

The MDA MET-derived cell lines HPSE-Low, HPSE-High, M225, and M343 were maintained in DMEM supplemented with 10% fetal bovine serum (FBS) at 37°C in sterile culture dishes as described previously.(12,29,32) All cell lines were *Mycoplasma-*free. Cells were subcultured by trypsinization in 0.5% trypsin (Sigma, St. Louis, MO) and 0.5 mM EDTA in Hank's balanced salt solution (HBSS) without calcium or magnesium in a laminar flow hood during their logarithmic phase of growth. For human osteoclastogenesis assays, 48-hour conditioned media (containing serum) from HPSE-High, HPSE-Low, M225, and M343 cells were collected, diluted 50% in α modified essential medium (α-MEM) and added to cultures of human peripheral blood mononuclear cells (as described below). We have shown previously that the 50% dilution of MDA MET-conditioned medium contains pro-osteoclastogenic activity, whereas 100% conditioned medium contains inhibitors that induce osteoclast progenitor death.(33)

### Heparanase activity assay

The heparanase activity assay used an immobilized [^3^H]heparan sulfate substrate and was performed as described previously.(11,34) Purified recombinant heparanase (46 ng; generously provided by Dr Hua Quan Miao, ImClone Inc., New york, NY) was used as the positive control, and buffer was used as the negative control. Each sample was normalized to equal volume and tested in triplicate on at least two separate occasions.

### IL-8 ELISA

The production and secretion of IL-8 was measured in conditioned medium using a commercially available enzyme-linked immunosorbent assay (ELISA) kit (R&D Systems, Minneapolis, MN, USA) according to the manufacturer's instructions. The commercially available IL-8 ELISA specifically detects human IL-8. IL-8 concentrations in conditioned media were calculated from a standard curve generated by adding recombinant IL-8 to the specific unconditioned medium and were considered undetectable if media concentrations were less than 0.3 pmol/L before correction for cell number. Murine NIH 3T3 cells tested with the IL-8 kit did not secrete IL-8 cross-reacting products. To block IL-8 activity, an IL-8 antibody (a kind gift of Dr Rakesh Singh, University of Nebraska, Omaha, NE, USA) was used as described previously.(32) Briefly, 200 µg/ml of IL-8 antibody was added every other day to osteoclast cultures. All IL-8 biologic activity is inhibited by this treatment.(33)

### Western blots

Cells were extracted, and Western blot analysis was performed using 60 µg of protein per lane and a mouse monoclonal antibody directed against recombinant human heparanase, as described previously.(11,12,34,35)

### Tumor biology

Four- to 6-week-old female SCID mice were purchased from Harlan (Indianapolis, IN, USA) and allowed to acclimate for 7 days. In the two experiments, animals (five or four per group) received up to 2 × 10^6^ cells (HPSE-Low or HPSE-High) in each of four different injection sites within the mammary fat pads (two axillary and two abdominal), as described previously.(12) All animal procedures were performed using a University of Arkansas for Medical Sciences (UAMS) IACUC-approved protocol.

### Histology and osteoclast determination

Osteoclasts were identified in mouse long bones that were excised, fixed in 10% neutral-buffered formalin for 2 days, and decalcified in 5% formic acid with agitation until deemed clear by the ammonium oxalate endpoint test.(36) The decalcified specimens then were dehydrated through graded ethanol and cleared in methyl salicylate before paraffin infiltration. Subsequently, the tissue was embedded in paraffin, sectioned (5 µm), and stained with hematoxylin and eosin (H&E) as described previously(32,36,37) and tartrate-resistant acid phosphatase (TRACP) using the Acid Phosphatase Leukocyte Kit (Sigma) as described previously.(32) Osteoclasts were identified as TRACP^+^ multinucleated cells apposed to the bone surface, as described previously.(33)

### Isolation and culture of peripheral blood mononuclear cells and their differentiation to osteoclasts

Peripheral blood was collected from healthy donors (approved by the UAMS Institutional Review Board) using heparin as an anticoagulant and 200 ng/mL RANK-Fc to minimize any priming of osteoclast progenitors by endogenous RANKL, as described previously.(32)

Blood was diluted in sterile PBS (1:1) in a sterile hood. The blood-PBS solution was slowly layered over AccuPrep solution (Accurate Chemicals, Westbury, NY, USA) and then centrifuged at 400*g* in swinging buckets for 30 minutes at 21°C. The peripheral blood mononuclear cell (PBMC) layer was collected and washed in five to six volumes of PBS, isolated by centrifugation at 140*g*, and resuspended in α-MEM containing 10% FBS. Cells were counted with a hemocytometer and plated in 48-well tissue culture plates at a concentration of 0.5 million cells in 0.5 mL volume per well. Macrophage colony-stimulating factor (mCSF, 25 ng/mL; R&D systems) was present in all treatment groups. RANKL (25 ng/mL; R&D systems) or IL-8 (10 ng/mL) were used as positive controls for the stimulation of osteoclastogenesis.(32)

Conditioned medium from heparanase-high, heparanase-low, M225, and M343 breast cancer cells growing in culture was added to wells that received only mCSF. For these experiments, medium was harvested 48 hours after cell plating, diluted 50% with α-MEM, and added to the cultures of human PBMCs. All conditioned-medium stimulation of osteoclastogenesis (and bone resorption) occurred in the absence of exogenously added RANKL or IL-8.

In addition, for some treatments, syndecan-1 was immunodepleted from conditioned medium using monoclonal antibody B-B4 (CD 138, Serotec, Raleigh, NC) and protein-G sepharose beads (GE Healthcare Bio-Sciences AB, Uppsala, Sweden). The levels of shed syndecan-1 accumulated in the conditioned media were assessed by ELISA using an Eli-pair kit from Diaclone (Cell Sciences, Inc., Norwood, MA, USA). The standard curve was linear between 8 and 256 ng/mL, and all samples were diluted to concentrations within that range, as described previously.(29)

Mononuclear cell cultures were maintained at 37°C, and half the medium in each well was replaced with fresh medium three times per week. The experiment was terminated on day 10. Medium was aspirated, and the cells were fixed with 10% formalin. TRACP staining was performed for quantitation of TRACP^+^ multinucleated cells. The number of osteoclasts present in the entire well was determined by manually counting TRACP^+^ cells that contained three or more nuclei. The numerical average of cell counts from four replicate wells per treatment was determined, and the results were expressed as the number of TRACP^+^ multinucleated cells per well per treatment group, as described previously.(32)

### PBMC-derived osteoclast cultures on dentine slices

Peripheral blood was collected from healthy donors, and PBMCs were isolated as described earlier. Dentine (a kind gift from Professor Tim Skerry, University of Sheffield, UK) was sliced into 0.5 × 0.5 cm pieces. The slices were collected in H_2_O and sonicated twice for 1 minute each to remove particle debris. They were then rinsed in two changes of water in between sonication and sterilized for at least 30 minutes in 100% ethanol. All dentine slices were stored in 100% ethanol until use. On the day of culture, dentine slices were washed four times with PBS and twice with α-MEM. Using sterile forceps, one slice was placed in each well of a 48-well plate (containing 0.5 mL of α-MEM), and the plate was incubated at 37°C for 30 minutes. Equilibration medium then was aspirated off, and PBMCs were added to the wells at a concentration of 1.0 × 10^6^ cells per well in 0.5 mL volume. Precursors were allowed to adhere to the slices for 4 hours at 37°C. Appropriate amounts of treatment medium were prepared, and 0.5 mL was added to wells in a replicate 48-well plate (lacking dentine slices) with 4 wells per treatment group. mCSF (25 ng/mL) was present in all treatment groups including control. The positive controls for stimulation of osteoclastic bone resorption was the addition of either RANKL (25 ng/mL) or IL-8 (10 ng/mL).(32) Using sterile forceps, slices (with adherent cells) were transferred to the second 48-well plate, being careful not to invert the slices. Half the medium was exchanged three times per week. Cells were allowed to grow on dentine slices for 10 to 12 days, after which time the cultures were terminated. Dentine slices were fixed in 10% formalin and stained for TRACP. The dentine slices then were mounted on glass slides and examined under a microscope. TRACP^+^ multinucleated cells showing the ability to resorb bone were counted as osteoclasts. Bone-resorption area was measured using histomorphometry software (Osteomeasure, Atlanta, GA, USA) after removal of the cells by sonication, as described previously.(32)

### Statistical analysis

Statistical significance between groups was determined by analysis of variance (ANOVA) prior to post hoc pair-wise multiple comparisons using the Student-Newman-Keuls method (SigmaStat, Aspire Software International, Ashburn, VA, USA); *p* < .05 was considered significant.

## Results

Breast cancer frequently metastasizes to bone via a process that is apparently facilitated by increased bone turnover.(6,38) In a previous study we demonstrated that primary breast tumor xenografts formed in the mammary fat pads of SCID mice expressing high levels of heparanase stimulated bone resorption with no evidence of any detectable tumor cells within the bone.(12) This study was performed to determine the mechanism for the distal osteolysis mediated by the heparanase-expressing tumor cells.

**Fig. 1:**
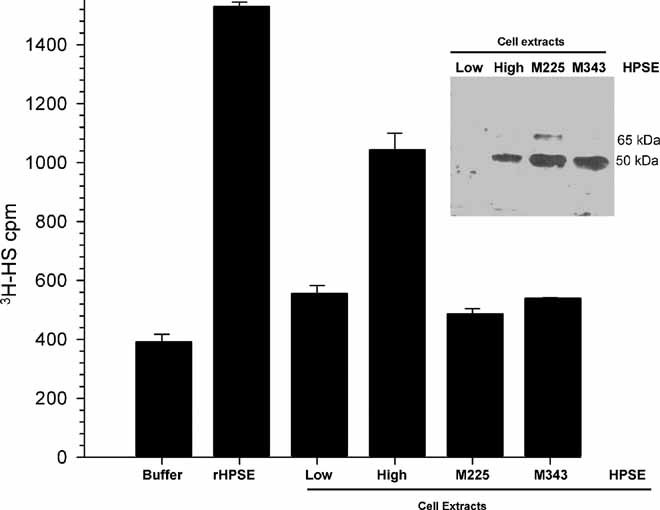
Heparanase protein levels and enzyme activity. The graph shows the heparan sulfate–degrading activity of the reaction buffer (Buffer), recombinant enzymatically active human heparanase (rHPSE, 1 µg), and cell extracts of HPSE-Low cells, HPSE-High cells, and cells expressing enzymatically inactive heparanase (M225 and M343). (*Inset*) Western blots of cell extracts probed with antibody to human heparanase. At the exposure shown, the most prominent band is the 50-kDa fully processed form of the enzyme in HPSE-High cells (High) and the two mutants (M225 and M343). Each lane was equivalently loaded for protein (30 µg).

Osteotropic MDA-MET breast cancer cells(3) were engineered to express high levels of wild-type heparanase (HPSE-High) or transfected with the empty vector (HPSE-Low).(12) In addition, MDA-MET cells expressing catalytically inactive heparanases designated M225 [mutated proton donor site of the active site (Glu225 to Ala225)] and M343 [mutated nucleophilic residue of the active site (Glu343 to Ala343)] were prepared. Analysis by Western blot and heparanase activity assays confirmed that cells overexpressing wild-type heparanase had high levels of heparanase protein and high levels of heparan sulfate degrading activity, whereas cells expressing mutant heparanases had high levels of heparanase protein that was catalytically inactive and did not degrade heparan sulfate(29) (Fig. 1).

**Fig. 2:**
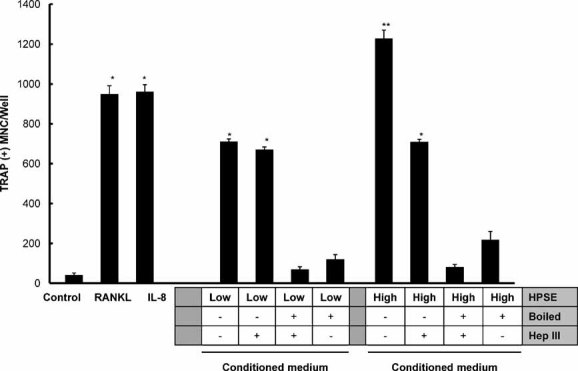
Conditioned medium from HPSE-High cells stimulates osteoclastogenesis. An in vitro osteoclastogenesis assay was used to evaluate conditioned medium from HPSE-Low (*left side*) and HPSE-High cells (*right side*), as indicated below the bars (HPSE). Other treatments of the medium prior to osteoclastogenesis (HepIII or boiled) are also indicated below the bars. Medium from HPSE-Low cells, as well as treatment with RANKL or IL-8, produced significant osteoclastogenesis compared with csf-1-only control (*p* < .05 indicated by single asterisk). Medium from HPSE-High cells was significantly higher than that of HPSE-Low cells (*p* < .05 indicated by double asterisk). HepIII-treated conditioned medium from HPSE-High cells reduced osteoclast formation to the extent that it was not significantly different from the osteoclastogenic activity of the conditioned medium from the HPSE-Low cells but was still higher than csf-1 control. Boiling the conditioned medium or boiling after HepIII treatment completely abolished the osteoclastogenesis activity of the conditioned medium from either the HPSE-High or HPSE-Low cells down to csf-1 control levels. Results are indicative of at least three replicate experiments.

Medium conditioned by HPSE-High cells enhanced osteoclastogenesis in vitro compared with medium conditioned by HPSE-Low cells, confirming our prior observations(12) (Fig. 2). Moreover, pretreatment of the conditioned medium with heparinase III (HepIII), a bacterial enzyme that extensively degrades heparan sulfate, completely abolished the enhanced osteoclastogenic activity of medium from HPSE-High cells (Fig. 2). This demonstrates that the enhanced osteoclastogenic activity of HPSE-High cells requires intact heparan sulfate. However, boiling the conditioned medium greatly reduced the osteoclastogenic activity of all samples regardless of the presence or absence of HepIII, demonstrating the importance of other protein factors in the conditioned medium (Fig. 2). Together these results suggest that the enhanced osteoclastogenic effect in the conditioned medium from HPSE-High cells requires both intact heparan sulfate chains and heat-labile factors.

**Fig. 3:**
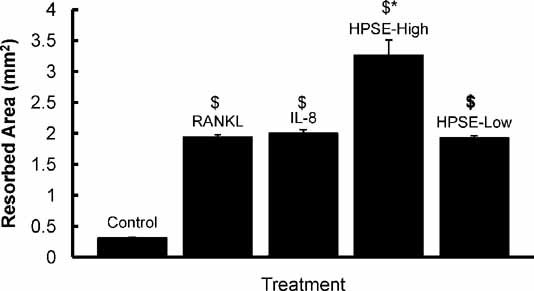
Medium from HPSE-High cells enhances bone resorption. Quantification of bone resorption on bone slices. Human osteoclasts were differentiated from PBMCs grown on bone and exposed to cell culture medium that was not conditioned by breast cancer cells (Control) or medium with addition of, RANKL (RANKL), IL-8 (IL-8), or medium conditioned by HPSE-High cells (HPSE-High), and medium conditioned by HPSE-Low cells (HPSE-Low). The $ indicates that all treatments are significantly different from csf-1 control (*p* *<* 0.05). The asterisk indicates that HPSE-High conditioned medium produced significantly more bone resorption than the all other conditions with *p* < .05. Results are indicative of at least two replicate experiments.

Next, osteoclasts were generated directly on human bone slices to measure the bone-resorbing activity of osteoclasts induced by medium from HPSE-High or HPSE-Low tumor cells. Consistent with the findings in Fig. 2, significantly more osteoclasts formed in response to medium from HPSE-High cells than medium from HPSE-Low cells (not shown), and the total area of bone resorbed by those osteoclasts was significantly greater than that in bone exposed to medium from HPSE-Low cells (*p* < .05; Fig. 3). However, osteoclasts generated in the presence of medium from HPSE-High cells created individual resorption pits that were not significantly different in area or depth from the pits formed by osteoclasts from HPSE-Low cells. In fact, the mean area of bone resorbed per osteoclast was 0.0036 ± 0.00067 mm^2^ for osteoclasts formed by medium from HPSE-high cells and 0.0032 ± 0.00053 mm^2^ for osteoclasts formed by medium from HPSE-Low cells, and these were not statistically different (*p* > .05). Similarly, no differences were observed in the number of nuclei per osteoclast in either HPSE-High or HPSE-Low conditioned-medium cultures (data not shown). Thus the enhanced bone resorption observed with the heparanase-expressing tumor cells is due to their impact on osteoclastogenesis (ie, increased osteoclast numbers) and not an effect of any increase in the activity or size of the individual osteoclasts.

**Fig. 4:**
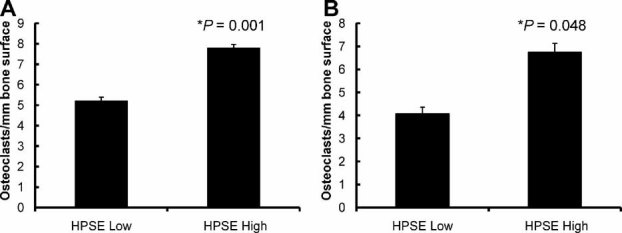
Orthotopic HPSE-High breast tumors increase osteoclast numbers in long bones. Femurs from animals bearing mammary fat pad tumors formed by HPSE-High or HPSE-Low cells were stained for TRACP, and the numbers of TRACP^+^ multinucleated cells were determined (*A*, experiment 1, *n* = 5 animals per group; *B*, experiment 2, *n* = 4 animals per group). A minimum of three sections of bone per animal were counted. Error bars represent standard error, and statistical significance was determined by a two-tailed *t* test. Significance was identified by *p* < .05.

Based on the results in Figs. 2 and 3, we hypothesized that the distal osteolysis induced by tumors in the mammary fat pad of mice(12) was due to increased osteoclast numbers in the long bones of mice bearing HPSE-High tumors compared with those from animals bearing HPSE-Low tumors. To test this, histomorphometric analysis of the long bones from tumor-bearing mice was performed. The results confirmed that significantly more osteoclasts per millimeter of bone surface were present in animals bearing tumors formed by HPSE-High cells compared with those bearing tumors formed by HPSE-Low cells (*p* < .05; Fig. 4). The animals with HPSE-High tumors also had increased serum levels of TRAP-5b and collagen telopeptides relative to levels of these markers in the sera of mice bearing tumors of HPSE-Low cells.(12) These data demonstrate that the increased osteoclast number observed in HPSE-High tumor-bearing mice is reflected in the increased serum biochemical markers of bone resorption.

**Fig. 5:**
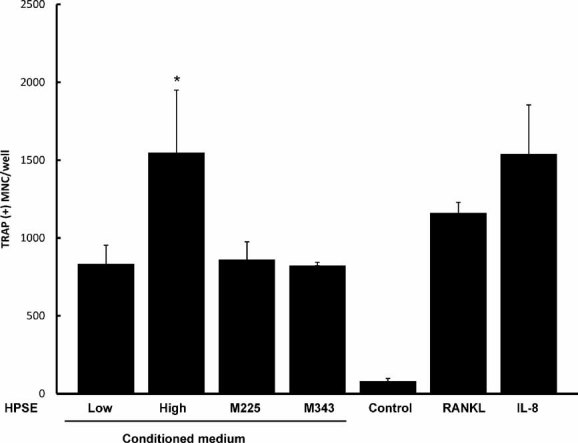
Enzymatically active heparanase is required for stimulation of osteoclastogenesis. Media conditioned by cells expressing enzymatically active or inactive heparanase were tested for the capacity to stimulate osteoclastogenesis in vitro. Medium from HPSE-Low cells induced osteoclastogenesis that was significantly greater than csf-1 control (*p < .05) and not different from M225, and M343 cells expressing inactive heparanases. The osteoclastogenic activity of HPSE-High cells was significantly greater than the other heparanase-expressing cells ($, *p* < .05). As a positive control, osteoclastogenesis induced following the addition of RANKL or IL-8 is also significantly different from control (*p < .05). Results are indicative of at least three replicate experiments.

Because enzymatically inactive heparanase also may exert effects on cell behavior.(9) the osteoclastogenic activity of medium conditioned by cells expressing enzymatically inactive heparanase was examined. The results demonstrated that medium conditioned by cells expressing enzymatically inactive heparanase does not enhance osteoclastogenesis beyond the level seen in medium from the HPSE-Low cells (Fig. 5).

In contrast to HepIII, which significantly degrades heparan sulfate into non–biologically active fragments, human heparanase cleaves heparan sulfate into biologically active fragments of 5 to 7 kDa.(9) In addition, heparanase also enhances the expression and shedding of syndecan-1 from the cell surface.(29) To determine whether heparan sulfate fragments or intact syndecan-1 were responsible for the increased osteoclastogenesis, syndecan-1 was immunoprecipitated from conditioned medium using the B-B4 antibody that recognizes the core protein of syndecan-1 (CD 138, Serotec, Raleigh, NC). This treatment removes all syndecan-1 from the medium to a level that is below the limit of detection of the syndecan-1 ELISA (≤17 ng/mL). In contrast, HPSE-High conditioned medium contained 43 ng/mL syndecan-1 after immunoprecipitation by beads coated with non-immune IgG.

**Fig. 6:**
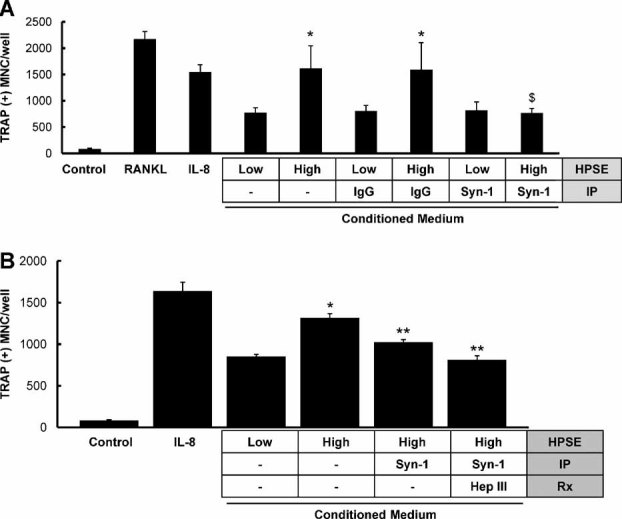
Shed syndecan-1 in conditioned medium of HPSE-High cells is required for enhanced osteoclastogenesis. (*A*) Osteoclastogenesis was increased significantly compared with control (csf-1 alone) in media conditioned by cells expressing low or high heparanase, as well as by the addition of IL-8 or RANKL (*p* < .05). Moreover, as expected, osteoclastogenesis was significantly higher for HPSE-high conditioned medium than for HPSE-low medium (*p < .05). However, only osteoclastogenesis stimulated by conditioned medium from cells expressing high levels of heparanase was decreased significantly by immunoprecipitation with a specific antibody to syndecan-1 (Syn-1, $) but not by immunoprecipitation with IgG. Results are indicative of at least three replicate experiments. (*B*) Immunoprecipitation of syndecan-1 from medium conditioned by heparanase-high cells significantly reduced the number of osteoclasts compared with medium without immunoprecipitation (**p < .05). Similalrly, immunoprecipitation of syndecan-1 from medium followed by HepIII treatment of medium also reduced the number of osteoclasts significantly compared with HPSE-High conditioned medium alone (**p < .05). HepIII treatment did not significantly reduce osteoclastogenesis compared with medium that lacked syndecan-1. Results are indicative of at least two replicate experiments.

The absence of syndecan-1 significantly diminished the enhanced osteoclastogenic activity of medium conditioned by HPSE-High cells (Fig. 6*A*). Immunodepletion and subsequent HepIII treatment further reduced the extent of osteoclastogenesis induced by the conditioned medium to the levels observed for HPSE-Low cells (Fig. 6*B*). Interestingly, the reduction in osteoclastogenic activity owing to HepIII treatment and syndecan-1 depletion was not significantly different from the osteoclastogenic activity of immunodepleted syndecan-1 alone. Collectively, these data demonstrate that shed syndecan-1, and not the heparanase-generated fragments of heparan sulfate, is critical for the enhanced osteoclastogenic activity of the tumors formed by the HPSE-High cells.

Elevated levels of the alpha chemokine IL-8 are associated with the increased bone metastasis and osteolysis of human breast cancer cells.(3,32,33) IL-8 is a heparan sulfate–binding osteoclastogenic factor and as such could cooperate with the heparan sulfate chains on the syndecan-1 shed by the HPSE-High cells. IL-8 is released into the medium by both HPSE-Low and HPSE-High cells. HPSE-High cells secrete 78 ± 2 ng/µg of IL-8 protein into conditioned medium compared with 32 ± 2 ng/µg secreted by HPSE-Low cells into the conditioned medium. Interestingly, IL-8 levels are also elevated in the sera of mice bearing tumors of HPSE-High cells (15 ± 2 ng/mL) compared with IL-8 levels in the sera of mice bearing HPSE-Low tumors (3 ± 1 ng/mL).

**Fig. 7:**
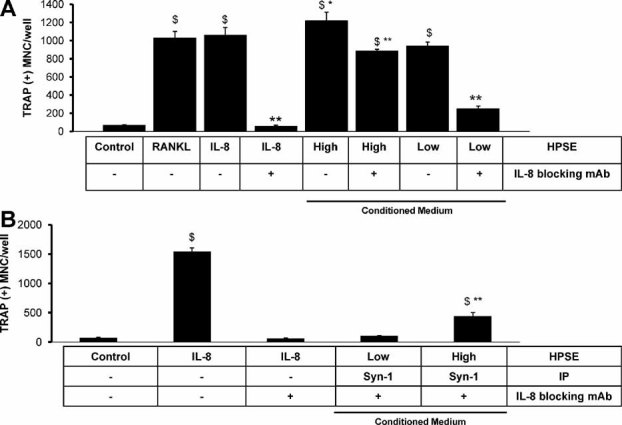
IL-8 is a heparan sulfate–binding cytokine that cooperates with shed syndecan-1 to promote osteoclastogenesis. (*A*) Addition of IL-8 or RANKL significantly increases osteoclast formation compared with csf-1 control, as does medium from HPSE-High or -Low cells ($, *p* < .05). Treatment with a neutralizing IL-8 antibody significantly decreased osteoclast formation in all groups (**) except RANKL. Treatment of heparanase-low cells with IL-8 antibody was significantly different from all other conditioned media groups (**p < .05). Conditioned medium from HPSE-High cells treated with IL-8 antibody retained significantly higher osteoclastogenesis than csf-1 control ($, *p* < .05). Addition of the IL-8 antibody significantly reduced the osteoclastogenesis relative to untreated HPSE-High conditioned medium (**p < .05); therefore, this data point is marked ($**). (*B*) Addition of IL-8 significantly increased osteoclast formation compared with csf-1 control ($, *p* < .05). The increased osteoclastogenesis was completely abolished by addition of the IL-8 function-blocking monoclonal antibody. Blocking IL-8 and depleting syndecan-1 dramatically reduced the osteoclastogenesis of both HPSE-Low and HPSE-High conditioned medium. The osteoclastogenic activity of HPSE-High conditioned medium was decreased significantly by IL-8 blockade and syndecan-1 depletion relative the IL-8-positive control (**) but remained significantly different from csf-1 control ($**). Results are indicative of at least two replicate experiments.

We and others have shown that IL-8 is a potent osteoclastogenic agent. Indeed, only 10 ng/mL of IL-8 is sufficient to stimulate osteoclastogenesis in in vitro preparations of PBMCs(33) (Fig. 7). A function-blocking antibody to IL-8 can completely abrogate the osteoclastogenesis mediated by recombinant human IL-8(33) (Fig. 7*A, B*; compare IL-8– to IL-8 + ). This IL-8 antibody also significantly suppresses the enhanced osteoclastogenic activity of the HPSE-High cells, demonstrating that IL-8 is an osteoclastogenic component of the conditioned medium (Fig. 7*A*). As expected, the same IL-8 antibody almost completely abrogated the osteoclastogenesis promoted by medium conditioned by HPSE-Low cells (Fig. 7*A*), demonstrating that IL-8 is the principal component of the osteoclastogenic activity in the medium from these cells. We investigated the combined effect of blockade of IL-8 and immunodepletion of syndecan-1 and found that the suppression of osteclastogenesis was greatly enhanced over either treatment alone (Fig. 7*B*).

## Discussion

This study addresses the mechanism(s) whereby expression of heparanase by tumor cells enhances systemic osteolysis. Using histomorphometric analysis of bones from an animal model of breast cancer and in vitro assays of osteoclastogenesis and bone resorption, we discovered that (1) mammary fat pad tumors expressing high levels of heparanase stimulate osteoclastogenesis in distant bones, (2) medium from cells expressing high levels of heparanase stimulates osteoclastogenesis in vitro and bone resorption at levels significantly above those of medium from cells expressing low levels of heparanase, and (3) the enhanced osteoclastogenesis requires the presence of syndecan-1, a proteoglycan whose shedding from tumor cell surfaces is enhanced by the expression of heparanase and protein agents, such as IL-8. In addition, these effects depend on the expression of the enzymatically active form of heparanase, suggesting that they occur downstream of the heparanase degradation of heparan sulfate. These results reveal a novel mechanism whereby expression of heparanase by tumor cells upregulates the shedding of syndecan-1, which after entering the systemic circulation stimulates osteoclastogenesis and enhances bone destruction.

The finding that syndecan-1 shed by breast cancer cells participates in driving osteolysis adds to the growing list of important functions of this proteoglycan within the tumor microenvironment. For example, the expression of syndecan-1 by stromal cells and its shedding have been linked to proliferation and angiogenesis of breast cancer cells, which is at least in part due to enhanced fibroblast growth factor 2 (FGF-2) signaling.(30,39) Increased shedding of syndecan-1 also has been associated with increased MCF-7 breast cancer cell invasion through matrigel, suggesting a role for shed syndecan-1 in the invasive phenotype of tumor cells.(40) Interestingly, shed syndecan-1 also promotes the growth, metastasis, and angiogenesis observed in multiple myeloma,(41) another cancer with an aggressive osteolytic phenotype.

Although our results demonstrate that the heparan sulfate chains of syndecan-1 are required for its effect in stimulating osteoclastogenesis, we cannot rule out the possibility that the core protein of syndecan-1 is also involved. This is particularly important in light of recent studies showing that the core protein of syndecan-1, even when added exogenously to cells, can activate αVβ3 and αVβ5 integrins.(24,25,42) Importantly, the αVβ3 integrin is expressed on the surfaces of osteoclasts,(43) and inhibitors of this integrin can block metastasis to bone and tumor-related osteolysis.(44,45) Thus it is compelling to speculate that the shed syndecan-1 activates the αVβ3 integrin on osteoclasts, thereby enhancing osteoclastogenesis.

The impact of shed syndecan-1 on osteoclastogenesis is important because it provides a novel mechanism for the extensive osteolysis seen in many breast cancer patients. Bone metastases frequently are associated with the later stages of disease progression and relapse and may not be evident when the cancer is first diagnosed,(46) suggesting that the initial stages of bone metastasis may not be completed when patients are initially diagnosed.

Bone turnover appears to be particularly important during the earliest stages of the formation of bone metastases(47,48) and is clearly involved in the initial colonization of bone by migrating breast cancer cells.(7) Once resorption is activated, disseminated tumor cells relocate to the bone marrow microenvironment and eventually the bone itself. As the metastatic foci grow, heterogeneity again may develop, potentially including cells with different metastatic capabilities and potential.(5)

Heparanase is upregulated in many tumor types and has been associated with increased metastasis and poor prognosis in breast cancer.(10) It has been suggested that much of heparanase function is regulated by its remodeling of the extracellular matrix and by its effects on cell signaling.(49) Our findings provide new insight into how heparanase regulates tumor behavior via upregulation of syndecan-1 shedding. Interestingly, enhanced syndecan-1 shedding depends on the heparan sulfate degrading activity of heparanase,(29) consistent with our finding that heparanase enzymatic activity is required for the enhanced osteoclastogenic effect of the heparanase-high breast cancer cells.

Overall, these results suggest a model where heparanase upregulation by tumor cells degrades heparan sulfate chains and enhances syndecan-1 shedding. The shed and active syndecan-1 is free to diffuse within the tumor microenvironment or to enter the circulation and travel to distal tissues, where it affects the behavior of host cells, such as osteoclasts and their precursors. It is also possible that the distal effects on osteoclasts we have observed helps to prepare premetastatic niches that are receptive to incoming circulating tumor cells. Further investigation of the role of the heparanase–shed syndecan-1 axis in breast cancer progression should focus on disruption of heparanase function, which is likely to profoundly affect tumor growth, metastasis, and osteolysis.

Immunodepletion of syndecan-1 from the medium is sufficient to reduce osteoclastogenesis to control levels, suggesting that syndecan-1 is the specific heparan sulfate proteoglycan that stimulates the enhancement of osteoclastogenesis by HPSE-High cells. In addition, the background (and substantial) osteoclastogenic activity of conditioned medium from HPSE-Low cells (and heparanase mutant cells) is presumably due to the expression of the osteolytic cytokine IL-8 that is still expressed by these cells.(33) Although syndecan-1 on the surfaces of myeloma cells can bind to osteoprotegerin (OPG), leading to its internalization and degradation,(50) we do not believe that this inhibits IL-8-mediated osteoclastogenesis because we have shown previously that the addition of OPG (or RANK-Fc) did not inhibit IL-8-induced osteoclastogenesis in vitro.(32,33) Moreover, in our in vivo model where we see enhanced osteoclastogenesis, the tumor cells are not present in the bone and thus are not likely altering local OPG levels in the bone. However, we cannot rule out the possibility that the clearance of OPG from the circulation by tumors distal to the bone could have some effect that contributes to the enhanced osteoclastogenesis in our animal model.

Collectively, these data demonstrate that shed syndecan-1 in the conditioned medium is not the sole agent stimulating osteoclastogenesis. Although syndecan-1 is the primary heparan sulfate proteoglycan stimulating osteoclastogenesis in the conditioned medium, boiling also abrogated osteoclastogenic activity. Heparan sulfate is impervious to boiling; however, proteins are not. This suggests that syndecan-1 acts in concert with other heparin-binding growth factors to stimulate the potent osteoclast formation observed. As mentioned earlier, an attractive candidate for this activity is IL-8. IL-8 is a CXC-chemokine that is activated by interaction with heparan sulfate(51) and that has been shown to directly stimulate osteoclast formation and activity.(32) Importantly, the heparanase-high cells were derived from the osteolytic IL-8 expressing MDA-MET human breast cancer cells.(3,12) As such, the heparanase-high (and heparanase-low) cells also produce significant and measurable levels of human IL-8 (HPSE-High: 78 ± 2 ng/µg of protein; HPSE-Low: 32 ± 5 ng/µg of protein). In addition, other heparin-binding growth factors may contribute to enhancing osteoclastogenesis or to creating an osteoclastogenic environment (e.g., FGFs, insulin-like growth factor–binding proteins, and platelet-derived growth factor). Binding of heparan sulfate of syndecan-1 to these factors can promote their interaction with high-affinity receptors on the cell surface and/or protect the factors from proteolytic degradation within the bone microenvironment. In addition, a region of the syndecan-1 core protein has been shown to activate αVβ3 integrin on cell surfaces.(25) Because activation of αVβ3 integrin on osteoclasts is critical for normal osteoclast function and bone resorption,(52) syndecan-1 also could be playing a role in stimulating osteoclastogenesis in vivo via the activity of its core protein interacting with osteoclast surface integrins.

Interestingly, after implantation in the mammary fat pad, serum IL-8 levels are measurable only in animals bearing HPSE-High tumors, suggesting a role for HPSE in the access of tumor-derived cytokines to the circulation (HPSE-High: 15 ± 2 ng/mL; HPSE-Low: 3 ± 1 ng/mL (at the detection limit of the assay). Understanding of the combined effects of heparanase, syndecan-1, and IL-8 on breast cancer progression, development of bone metastases, and activation of bone resorption is currently the focus of intense investigation in our laboratory. Whatever the case, it is clear that the osteolytic phenotype of aggressive human breast cancer cells involves multiple factors, including, but not limited to, heparanase, syndecan-1, and IL-8.

The finding that syndecan-1 shed from the surface of tumor cells is another important mediator of osteoclastogenesis identifies the heparanase–syndecan-1–cytokine axis as a novel target for inhibiting osteoclastogenesis and ultimately for preventing the consequences of bone metastases. We and others have speculated that heparanase acts as a master regulator of the aggressive tumor phenotype(29) that is likely the result of heparanase effects on multiple cell behaviors, including the regulation of tumor osteolysis. Further investigation of the role of this important axis in breast cancer progression should focus on disruption of heparanase function, which is likely to profoundly affect tumor growth, metastasis, and osteolysis.
